# Expression of Das-1, Ki67 and sulfuric proteins in gastric cardia adenocarcinoma and intestinal metaplasia lesions

**DOI:** 10.3892/etm.2013.1038

**Published:** 2013-04-02

**Authors:** XIAO-SHAN FENG, YU-FENG WANG, SHU-GUANG HAO, YI RU, SHE-GAN GAO, LI-DONG WANG

**Affiliations:** 1 Department of Oncology, Cancer Institute, The First Affiliated Hospital of Henan University of Science and Technology, Luoyang, Henan 471003;; 2 Henan Key Laboratory for Esophageal Cancer Research, College of Medicine, Zhengzhou University, Zhengzhou, Henan 450052;; 3 Department of Thoracic Surgery, Xinxiang Center Hospital, Xinxiang, Henan 453000, P.R. China

**Keywords:** Das-1, Ki67, gastric cardia adenocarcinoma, intestinal metaplasia, histochemistry, immunohistochemistry

## Abstract

The aim of this study was to characterize histo-chemical patterns and Das-1 and Ki67 protein expression in gastric cardia adenocarcinoma (GCA) and intestinal metaplasia (IM) lesions adjacent to GCA. Histochemical techniques, including Alcian blue/periodic acid-Schiff (AB/PAS), high iron diamine/Alcian blue (HID/AB) and avidin-biotin-peroxidase complex (ABC) immunohistochemistry were applied to GCA and IM samples from patients (n=200) in Linzhou, Henan, China, a high incidence area for GCA and esophageal squamous cell carcinoma (SCC). The detection rate of IM lesions in resected tissues adjacent to GCA was 32.5% (65/200). GCA and IM lesions presented a high frequency of Das-1 and Ki67-positive staining with statistical significance (P<0.01). The expression of sulfuric proteins did not show co-expression with Das-1 and Ki67 in GCA and surrounding IM lesions (P>0.05) from the same GCA patient. The high frequency of co-expression of Das-1 and Ki67 in GCA and adjacent IM lesions indicates that IM adjacent to GCA may undergo similar molecular changes to GCA, which may be one of the mechanisms for malignant transformation of IM in the population studied.

## Introduction

The epidemiology of gastric cardia adenocarcinoma (GCA) is characterized by a regional distribution identical to that of esophageal squamous cell carcinoma (SCC) (1). In Linzhou, northern China, the ratio of the incidence rates for GCA and SCC is ∼1:3.2 (2). Approximately 60% of patients with suspected SCC, who receive surgical treatment at Linzhou People's Hospital, are confirmed as having preliminary SCC and the remaining 40% have GCA (3). Furthermore, it is not a rare event in other high incidence areas for esophageal cancer (4). An epidemiological study has suggested that SCC and GCA share a similar pathogenesis of malignant transformation (5). The occurrence pattern of GCA is not similar to that of neoplasms originating from the distal parts of the stomach. In recent years, the incidence of gastric cancer has been declining (6), while that of GCA is tending to increase (7). In reference to variations in the epidemiology, etiology, pathophysiology and clinical parameters between GCA and cancer in the distal part of the stomach, we have proposed that GCA should be regarded as an independent disease (8,9).

Like other forms of cancer, GCA is a multi-step and progressive disease, which may require the involvement of the alterations of multiple genes (10). The resulting imbalance of homeostasis between tumor suppressor and oncogene is one of the key elements in this chronic disease (11,12). Although intestinal metaplasia (IM) is an inevitable step in the histopathological model for GCA, which begins with normal mucosa and progresses towards superficial cardia gastritis, atrophic cardia gastritis and/or interstitial metaplasia, dysphasia and finally to GCA (13), the underlying molecular mechanisms for this multi-step process are not clear. The present study aimed to examine the expression of sulfuric, Das-1 and Ki67 proteins in GCA and IM adjacent to GCA to enhance our understanding of the correlation between IM and GCA.

## Materials and methods

### GCA patients and sample collection

Surgically resected GCA samples from 200 patients (including 147 males and 53 females with a mean age of 59±9 years and an age range of 46–79 years) were collected from Linzhou People's Hospital, Linzhou Central Hospital and Esophageal Cancer Hospital of Yaocun, Linzhou in 2010. This study was conducted in accordance with the Declaration of Helsinki. This study was conducted with approval from the Ethics Committee of The First Affiliated Hospital of Henan University of Science and Technology. Written informed consent was obtained from all participants. No chemotherapy or radiotherapeutic regimens were undertaken for the patients involved in this study. All the samples were fixed with 95% ethanol. In addition to GCA tissue blocks, 10–15 tissue blocks adjacent to the GCA field were also dissected and then subjected to paraffin-embedding. Serial sections of 5 μm were cut for hematoxylin and eosin (H&E) staining, histochemistry and immunohistochemistry (IHC).

### Histochemical staining

High iron diamine-Alcian blue (HID/AB) staining was performed according to the protocols established in our laboratory previously (14). In brief, deparaffinized sections were treated with high iron diamine for 24 h at room temperature followed by incubation with Alcian blue solution for 20–30 min in a humified chamber. After washing with distilled water, sections were treated with 0.5% neutral red solution (Shanghai No. 3 Reagent Factory, Shanghai, China) for 1–2 min and then washed as above. The slides were sealed with neutral gum. As for the avidin-biotin-peroxidase complex (ABC) method of IHC, slides were deparaffinized with xylene and dehydrated with serially graded ethanol followed by antigen retrieval by microwave boiling for 10 min. After washing with phosphate-buffered saline (PBS) three times for 5 min each, the slides were incubated with 0.5% H_2_O_2_ for 20 min to quench endogenous peroxidase. Normal horse serum at a dilution of 1:50 was added to each slide to block non-specific reactions and incubated for 20 min. Incubation with the primary mouse antibodies for Das-1 (Uscn Life Science Inc., Wuhan, China) and Ki67 (Oncogene Research, San Diego, CA, USA) was performed at 4°C overnight. The dilution of Das-1 was 1:50. Following incubation with biotinylated rabbit secondary antibody at a dilution of 1:200 for 45 mins and three washes with PBS, ABC solution at a dilution 1:50:50 was applied to each slide and incubated for 1 h. The positive results were visualized with 3,3′-diaminobenzidine (DAB). Finally, slides were mounted with neutral gum. During each batch of experiments, the control slide (without the primary antibody) was used to ensure the protocols were followed correctly.

### Criteria for HID/AB and IHC analyses

The criterion for diagnosis using HID/AB has been established previously (15). The sulfuric mucous was detected as black staining while the sialic mucus appeared as blue staining in the cytoplasm and/or outside the cells under a microscope. For positive Das-1 staining, the cytoplasm presented brown staining and there was no staining in the nuclei. In accordance with Mirza *et al*(16), the expression of Das-1 was considered positive if a substantial number of cells and >1 gland were stained. If only an occasional goblet cell was stained, the sample was considered negative. With regard to the criterion for Ki67 staining, brown staining was observed exclusively in the nuclei of cells. Ki67 staining was classified according to the protocols reported by Yi (17): + represents <25% positively-stained cells, ++ indicates 26–50% positive cells, +++ indicates 51–75% positive cells and ++++ indicates >76% positively-stained cells in ten randomly selected fields under a microscope with ×400 magnification.

### Statistical analysis

The κ consistency test was employed to evaluate the significance of statistical analyses. P<0.05 was considered to indicate a statistically significant difference.

## Results

### Detection rate of IM lesions adjacent to GCA

The detection rate of IM lesions in resected tissues adjacent to GCA was 65/200 (32.5%).

### Ki67 protein expression in GCA and matched IM lesions

**Figure 1:**
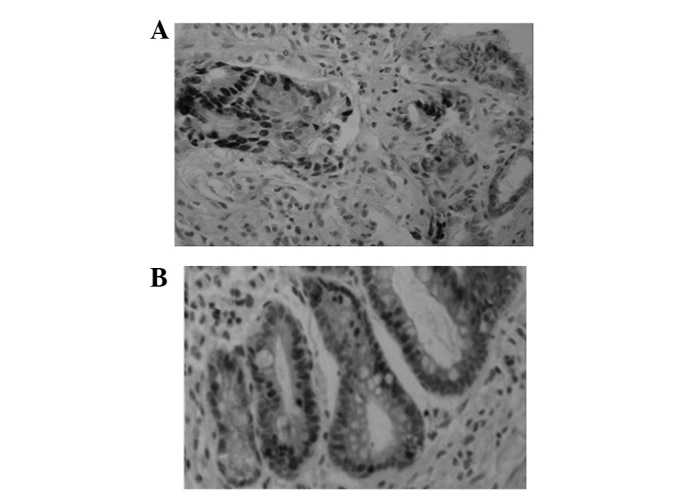
(A) Ki67 immunostaining of the gastric cardia adenocarcinoma (GCA). The nuclei demonstrated strong immunoreactivity (magnification, ×10). (B) Ki67 immunostaining of intestinal metaplasia lesions adjacent to the GCA. The nuclei demonstrated strong immunoreactivity (magnification, ×20).

Ki67 expression was identified in GCA and in the adjacent IM of the same patient (Fig. 1 and Table I).

### Das-1 protein expression in GCA and matched IM lesions

**Figure 2:**
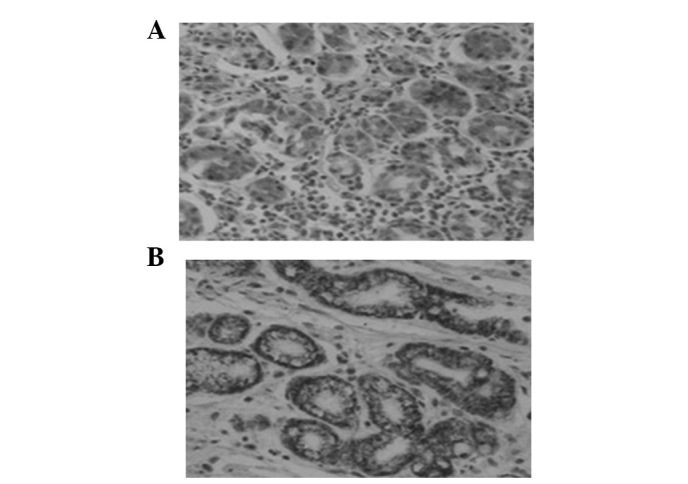
(A) Das-1 immunostaining of the gastric cardia adenocarcinoma (GCA). The positive immunoreactivity was located in the nuclei (magnification, ×10). (B) Das-1 immunostaining of intestinal metaplasia lesions adjacent to the GCA. Positive immunoreactivity was located in nuclei (magnification, ×20).

Similar to the expression pattern of Ki67, the Das-1 protein was identified in GCA and the adjacent IM of the same patient (Fig. 2 and Table I).

### Sulfuric mucous protein in GCA and matched IM lesions

**Figure 3:**
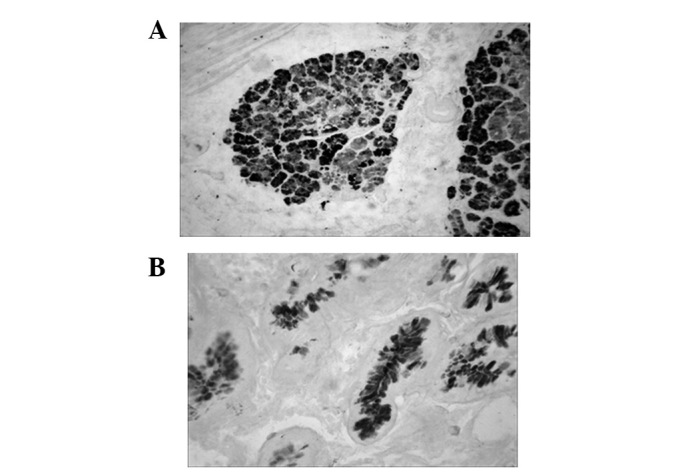
(A) Histochemical stain for sulfuric protein in gastric cardia adeno-carcinoma (GCA). The sulfuric mucous was detected as black staining, while the sialic mucous appeared as blue staining in the cytoplasm (magnification, ×20). (B) Histochemical stain for sulfuric protein in intestinal metaplasia (IM) lesions. The sulfuric mucous was detected as black staining, while the sialic mucous appeared as blue staining in the cytoplasm (magnification, ×20).

The sulfuric mucous protein was stained as black-brown in GCA following HID/AB staining. In the same patient, the sulfuric mucous protein did not show a pronounced co-expression tendency in GCA and surrounding IM tissues (Fig. 3 and Table I).

## Discussion

The current study demonstrates the presence of Das-1 protein in GCA and the surrounding IM lesions. This finding implies that these two morphologically different lesions are homologous in terms of molecular alterations. GCA may originate from IM. Das-1 protein may contribute to the transformation from IM to GCA in this multi-stage process. Another finding of the present study was the co-expression of the Ki67 protein in GCA and the corresponding IM, which further supports our above hypothesis.

Das-1 (formerly known as 7E12H12) was developed against a 40-kDa colonic epithelial protein (18). It specifically recognizes a >200-kDa colon epithelial protein that complexes with a 40-kDa protein and acts as a chaperone to bring the 40-kDa protein to the colonic epithelial surface. Its role in cell proliferation, differentiation and carcinogenesis is unclear. Experimental data show that high Das-1 protein expression may be used to identify risk factors for the occurrence of cancer of cardia intestinal tissue.

The colonic type of IM, which contains sulfuric mucous protein, has more malignant features and has increased malignant potential. Morphologically, colonic IM is immature and/ or dedifferentiated with respect to histological structure and cell appearance. This type of IM is considered a manifestation of a typical cardia mucosa. The presence of a large quantity of sulfuric mucous protein in the glands is often indicative of aberrant gene expression. In IM lesions, a variety of tumor-associated antigens, including the Ras p21 oncogene product, are increasingly identified. In terms of genetic features, aneuploidal cells and the DNA content in the colonic type of IM increase with IM progression. Increasing evidence indicates that the colonic type of IM shares more biological characteristics with neoplastic cells than other type of IM (19). The present study also revealed that the detection rate of the colonic type of IM adjacent to GCA was increased compared with that in normal cardia mucosa. The sulfuric mucous protein, however, did not show the consistency as strikingly as Das-1 and Ki67 proteins, which demonstrate pronounced co-expression. The possible causes for the different expression patterns of sulfuric mucous protein may be as follows: i) IM containing sulfuric mucous protein, although readily prone to be transformed, has to undertake a multi-step process and clone selection prior to gaining malignant properties; ii) the number of samples involved in this study is limited and, therefore, a larger study is required; and iii) the correlation between the colonic type of IM and GCA requires in-depth investigation.

Currently, the mechanisms of carcinogenesis in the IM mucosa are not clear. The epithelial cells in IM and the enzymes within are able to take up lipids and lipid-soluble substances, in a similar manner to the intestinal epithelium; however, the epithelium of IM does not possess infrastructure-like chyle vessels for transporting these substances. These retained substances inside IM cells are highly carcinogenic and may contribute to the transformation (20). Previously, it was hypothesized that genes of stem cells in the proliferation center within a mucosal gland are mutated following stimulation, and the depression of colonic types of gene leads to the differentiation of stem cells towards colonic-type cells. Two types of IM, complete IM and incomplete IM, are formulated based on the mature or immature morphology of metaplasia. The lesions are likely to get worse if differentiation is aggravated and ultimately form a neoplasm (21).

In conclusion, these results not only increased our insight into the correlation between IM and GCA using histochemistry and IHC, but also provide a basis for the elucidation of the tumor-genesis mechanisms of GCA and the identification of highly-specific and highly-sensitive biomarkers for detection and early diagnosis. In combination with clinical practice, further studies are required to determine the importance of histochemical staining in the classification of GCA, to examine the correlation between grades of malignancy, lymphatic metastasis and various types of IM and to investigate the response sensitivity following radiotherapy and chemotherapy.

## Figures and Tables

**Table I:** Co-expression of Das-1, Ki67 and sulfuric proteins in GCA and matched adjacent IM lesions from the same patient.

		GCA								
		Das-1 protein			Ki67 protein			Sulfuric protein		
		Positive n (%)	Negative n (%)	Total	Positive n (%)	Negative n (%)	Total	Positive n (%)	Negative n (%)	Total
IM (adjacent to GCA)	Positive	22 (48.75)	3 (3.75)	25	20 (30.77)	2 (3.08)	22	19 (29.23)	19 (29.23)	38
	Negative	9 (5.00)	31 (42.50)	40	19 (29.23)	24 (36.92)	43	10 (15.38)	17 (26.15)	27

Das-1: κ=0.503, P<0.01; Ki67: κ=0.436, P<0.01; sulfuric protein: κ=0.129, P=0.322. GCA, gastric cardia adenocarcinoma; IM, intestinal metaplasia.
